# Antioxidant and Cytotoxicity Activities of Karamunting (*Melastoma malabathricum L.*) Fruit Ethanolic Extract and Quercetin

**DOI:** 10.31557/APJCP.2019.20.2.639

**Published:** 2019

**Authors:** Isnaini Isnaini, Alfi Yasmina, Hendra Wana Nur’amin

**Affiliations:** *Department of Pharmacology and Therapeutics, Faculty of Medicine, Lambung Mangkurat University, South Kalimantan, Indonesia. *

**Keywords:** M. malabathricum L, quercetin, kaempferol, antioxidant, cytotoxicity

## Abstract

*Melastoma malabathricum L.* is a type of plant naturally grows in Kalimantan that has medicinal properties. Ethanolic extract of *M. malabathricum L.* flower has quercetin and kaempferol contents that have antioxidant and anticancer activities. But the antioxidant and cytotoxicity activities of *M. malabathricum L.* fruit ethanolic extract were not known. This study measured the quercetin and kaempferol level in *M. malabathricum L.* fruit ethanolic extract using HPLC MS/MS, antioxidant activity using DPPH method, and cytotoxicity activity using Brine Shrimp Lethality Test (BSLT) method. Results showed the level of quercetin and kaempferol from *M. malabathricum* L fruit ethanolic extract are 67.78 µg/g and 43.52 µg/g, respectively. Beside, the antioxidant activity with by IC50 was 16.82±0.24 ppm and 7.38±0.41 ppm. The cytotoxicity activities of *M. malabathricum* L. fruit ethanolic extract and quercetin are shown by the LC50 of 313.44 ppm (95%CI 283.97-344.43) and 37.24 ppm, respectively.

## Introduction

*Melastoma malabathricum L.* is a natural plant found in Kalimantan, which is used for traditional drug treatment. It grows wildly in the area and has not been used optimally. Several pharmacological activities of *M. malabathricum* have been reported, namely, antibacterial (Sunilson et al., 2008; Choudhury et al., 2011; Isnaini et al., 2011), antidiarrheal (Nur’amin et al., 2010; Sunilson et al., 2009), antioxidant (Susanti et al., 2007; Chalise et. al., 2010), gastroprotective (Al-Bayaty et. al., 2008), wound healing (Simanjuntak, 2008; Sunilson et al., 2008), antinociceptive (Sulaiman et al., 2004; Zakaria et al., 2006), anticoagulant (Manicam et al, 2010), antiinflammatory (Mazura et al, 2007; Zakaria et al., 2006), antiviral (Nazlina et al., 2008), and anticancer activities (Susanti et al., 2007; Nazlina et al., 2008).

Parts of the plant that can be used for treatment include the flower and the fruit of *M. malabathricum *L. Its flower is known to contain quercetin and kaempferol (Isnaini et al., 2018). Quercetin and kaempferol are natural polyphenols that have multiple antioxidant properties that may eliminate ROS through free radical scavenging, metal ion chelation, pro-oxidant enzyme inhibition, antioxidant activation, and enzyme detoxification (Amic and Lucic, 2010). Both compounds can react directly with oxidants and also can induce endogenous antioxidants, such as MnSOD. Quercetin and kaempferol are known to be able to cleave Nrf2 and keap 1, so that Nrf2 is activated and may induce MnSOD (Kimura et al., 2009). Quercetin induces MnSOD in the hepatoma cell line (Amic and Lucic, 2010). Kaempferol induces MnSOD and CAT proteins in human non-small cell lung carcinoma cell line (H460) (Leung et al., 2007). 

Quercetin and kaempferol also have anticancer activity. Quercetin effect as anticancer depends on the concentration. Quercetin with the concentration of 1-20 µM has an antiproliferative effect, but in the concentration of 50-200 µM has proapoptotic effect by decreasing total viable cells through increased cell apoptosis (Jaganathan and Mandal, 2009). Kaempferol increases cell growth at low concentration (1-10 µM) by being estrogen receptor agonist and increasing DNA synthesis. However, kaempferol inhibits DNA synthesis cell growth of MCF-7 cell line at the concentration of 20-90 µM (Wang and Kurzer, 1997). Quercetin causes an increase in phosphatase and tensin homolog (PTEN) and p27, and a decrease in Akt, so that it inhibits growth and causes apoptosis (Kok et al., 2008). Quercetin and kaempferol are known to impair cell cycle at G1/S and G2/M checkpoints (Chahar et al., 2011).

*M. malabathricum *L. fruit is assumed to contain quercetin and kaempferol, as in *M. malabathricum *L. flower. Until recently, quercetin and kaempferol contents in *M. malabathricum *L. fruit antioxidant and cytotoxicity effect of *M. malabathricum *L. fruit extract were not known. This study aimed to analyze the antioxidant and cytotoxicity effect of *Melastoma malabathricum L.* fruit extract.

## Materials and Methods

*Collection of M. malabathricum L. fruit samples*

This study used *M. malabathricum *L. fruit. The fruits were collected in the morning before 10.00 AM from Gunung Kupang area, Banjarbaru, South Kalimantan (3028’39.02” S and 114051’18.69” E). This plaint was identified in the Laboratory of Basic Sciences, Lambung Mangkurat University, Banjarbaru, South Kalimantan, Indonesia.

*Extraction of M. malabathricum L. fruit*

*M. malabathricum *L. fruit was extracted using the maceration method. Pulverized *M. malabathricum *L. fruit was soaked in ethanol 96% solvent for 24 hours, and occasionally stirred. Extraction was conducted until the solvent was colorless. The extract was obtained by evaporation using rotary evaporator until thick extract was obtained. It was freeze dried to acquire dry powder.

*HPLC analysis of quercetin and kaempferol contents in M. malabathricum L. fruit*

Quercetin and kaempferol contents were. analyzed with HPLC-MS/MS. The columns used were from Hypersil Gold specification (50 mm x 2.1 mm x 1.9 µm). UHPLC (ACCELLA type 1,250 from Thermo Scientific) consists of vacuum degasser, quaternary pump, thermostatic autosampler, that were controlled with a personal computer using X-Calibur 2.1 program. Mobile-phase A consisted of 0.1% formic acid in aquabidest, and phase B consisted of 0.1% formic acid in acetonitrile. A linear gradient with the rate of 300 µL/min with mobile phase management as follows: 0-0.6 minutes: 15% B, 2-3.5 minutes 100% B, and 4.5 minutes 15% B. Injected volume in LC was 2 µL. The column was maintained at 30°C, and autosampler compartment was maintained for 10°C. Standard calibration for kaempferol with precursor ion 285 m/z produced transitional ions 239, 229, 255 mz. Standardization of quercetin precursor ion was 301 m/z produced transitional ions 170, 245, 272 m/z.

Determination of quantity with the Selected Reaction Monitoring (SRM) method was controlled at 301>179 m/z for quercetin and at 285>229 m/z for kaempferol. Ionization condition of ESI was as follows: spray pressure 3 kV, evaporation temperature 270°C, capillary temperature 300°C, nitrogen as sheath gas pressure 40 psi, and Aux gas pressure 10 psi with Argon gas.

$\mathrm{Activity}=\frac{(\mathrm{A for blank}-\mathrm{A for sample}}{\mathrm{A for blank}}\times 100\%$

*Antioxidant activity*

The extract was made into several concentration in ethanol at several concentrations (5-25 ppm) for 1 ml, and then 3 ml DPPH 40 ppm in ethanol was added. As the control, 1 ml ethanol was added with 3 ml DPPH 40 ppm. They were left in a dark room for 30 minutes, then the absorbance was measured at λ 517 nm.

**Table 1:** Antioxidant and Cytotoxic Activities of *M. malabathricum L*. and Quercetin

Compound	Antioxidant activity	Cytotoxicity
	IC50 ± RPD	LC50 (95% CI)
*M. malabathricum L.* fruit extract	16.82 ± 0.24 ppm	313.44 ppm (283.97-344.43)
Quercetin	7.38 ± 0.41 ppm	37,24 ppm (n.a.)

**Figure 1:**
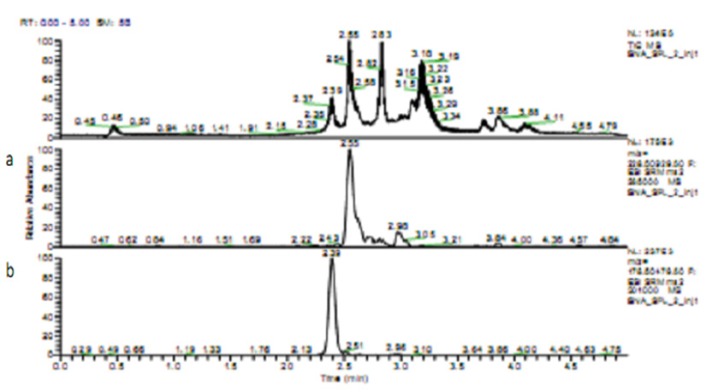
Chromatogram of Kaempferol (a) Quercetin (b) in Ethanolic Extract of *M. malabathricum L*. Fruit

**Figure 2:**
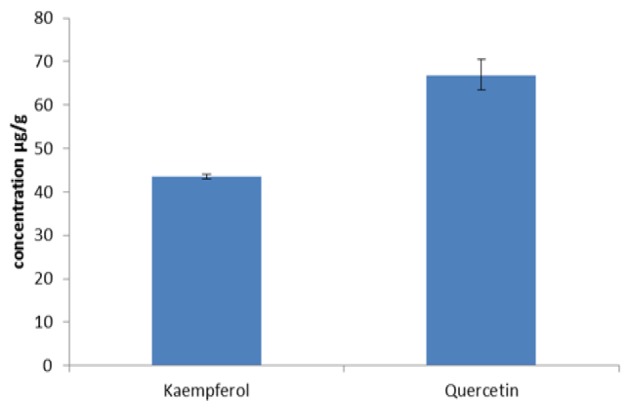
Concentrations of Kaempferol and Quercetin in *M. malabathricum L*. Fruit Extract Measured with HPLC

Antioxidant activity (by capturing DPPH radicals) data for ethanolic extract of *M. malabathricum* L. fruit and the pure quercetin were then analyzed and the IC50 values were estimated with probit analysis with Vitamine C as the comparator.

*Cytotoxicity test*

One of the methods to find out anticancer activity is by conducting cytotoxicity test in brine shrimp, usually called Brine Shrimp Lethality Test (BSLT). Brine shrimp embryos were incubated using artificial sea water with 35% salinity. The containers were filled with ±50-100 brine shrimp eggs, and then they were left for 48 hours. Ethanolic extract of *M. malabathricum *L. fruit was diluted with DMSO 0.05% in 35% salinity sea water to create the concentrations of 62.5, 125, 250, 500, 750, and 1,000 ppm. A total of 20 ml of each solution was put into each container. Each container containing 10 brine shrimps. The control was sea water with 35% salinity added with DMSO 0.05%. The containers were left for 24 hours, and then the total numbers of dead and survived brine shrimps were calculated from each container, and probit analysis was used to estimate the LC50.

## Results

*Measurement of quercetin and kaempferol in ethanolic extract of M. malabathricum L. fruit*

The measurement of quercetin and kaempferol with HPLC showed that the quercetin content of the extract was 67.78 µg/g and the kaempferol content was 43.52 µg/g (Figure 1 and 2). HPLC results showed that quercetin had a higher concentration in the extract compared to kaempferol. In the next steps, ethanolic extract of *M. malabathricum* L. fruit was compared only to quercetin.

*Antioxidant and cytotoxic activities of ethanolic extract of M. malabathricum L. fruit and quercetin*

Ethanolic extract of *M. malabathricum *L. fruit has lower antioxidant activity compared to quercetin (IC50 16.82 ± 0.24 ppm vs 7.38 ± 0.41 ppm). Cytotoxicity test with BSLT method showed that ethanolic extract of *M. malabathricum *L. fruit had higher LC50 compared to quercetin (313.ppm [95%CI 283.97-344.43] vs 37.24 ppm [95%CI n.a.]) (table 1). This means that ethanolic extract of *M. malabathricum *L. fruit had lower cytotoxicity activity compared to quercetin. 

## Discussion

Based on this study, ethanolic extract of *M. malabathricum* L. fruit contains quercetin (67.78 µg/g) and kaempferol (43.52 µg/g). Quercetin content in the fruit is higher than kaempferol, which is different from other studies on quercetin and kaempferol contents in ethanolic extract of *M. malabathricum *L. flower. In various phases of flowers, kaempferol content is higher than quercetin (Isnaini et al., 2018). Quercetin and kaempferol are biosynthesized from narigenin. Narigenin is converted to dihydrokaempferol, and then to kaempferol. Dihydrokaempferol may also be converted to dihydroquercetin, that eventually will be converted to quercetin (Awang et al., 2012). The pathway to produce kaempferol is shorter than that for quercetin; therefore in the flower there is more kaempferol content compared to quercetin (Awang et al., 2012), but in the fruit, it occurs otherwise.

Antioxidant activity of ethanolic extract of *M. malabathricum *L. fruit is weaker than that of quercetin. Many compounds contained in ethanolic extract of *M. malabathricum *L. fruit affect its antioxidant activity. Aside from quercetin and kaempferol, ethanolic extract of *M. malabathricum *L. fruit may also contain naringenin (Susanti et al., 2007), anthocyanin (Abdullah et al., 2006) and malvidin-3,4-diglucosyde (Joffry et al., 2012), which also have antioxidant activity (Kharadze et al., 2018; Zaidun et al., 2018). Meanwhile, ethanolic extract of *M. malabathricum *L*.* fruit with antioxidant IC50 of 16.82 ppm would have 1.14 ppm quercetin. This IC50 of quercetin in *M. malabathricum *L. fruit extract is smaller than the IC50 of pure quercetin to achieve the same antioxidant activity.

Cytotoxic activity of *M. malabathricum *L. fruit extract was lower than that of quercetin (LC50 313.44 ppm vs 37.24 ppm). Ethanolic extract of *M. malabathricum *L*.* fruit with LC50 of 313.44 ppm would have quercetin content of 67.78 µg/g extract, namely 21.24 ppm quercetin. This LC50 of quercetin in *M. malabathricum *L*.* fruit extract is smaller than the LC50 of pure quercetin to achieve the same cytotoxic activity. Naringenin and anthocyanins found in M. melastoma L. fruit also have anticancer activity (Lee et al., 2018). This means that there are other compounds in the fruit extract that have synergistic antioxidant and cytotoxic activities.

Quercetin is the strongest flavonoid to protect the body from reactive oxygen species. Free radicals can impair cellular function and eventually leads to cell death. Quercetin can become direct and indirect antioxidants. Quercetin acts as a direct antioxidant by scavenging free radicals. As an indirect antioxidant, quercetin can induce antioxidant response element (ARE) by activating Nrf2 (Kimura et al., 2009; Miyamoto et al., 2011). Nrf2 is known to be able to induce endogenous antioxidant, namely, CAT, SOD, and GPx (Gibellini et al., 2010). Based on the research by Chang et al., (2009), quercetin can induce MnSOD in human hepatoma cell line HA22T/VGH at the concentration of 40 µM and 60 µM after 48 hours exposure.

Ethanolic extract of *M. malabathricum *L. fruit containing quercetin and kaempferol have lower antioxidant and cytotoxic activities compared to pure quercetin. These linear antioxidant and cytotoxicity activities may be due to the role of oxidants and antioxidants on cell apoptosis and proliferation. In the study by Isnaini et al., (2018) in breast cancer cell line MCF-7, high oxidants and antioxidants, particularly superoxide radicals, lead to cell proliferation. Therefore the lower antioxidant effect, the lower cytotoxicity from the extract.

Superoxide radical is one of ROS family. ROS can cause increased proliferation, inhibition of proliferation and cell apoptosis depending on its level. The increase in cell numbers is due to an increase in cell proliferation. Cell proliferation occurs through the Akt pathway. ROS may inhibit PTEN resulting in a continuous cycle of the cell path despite mutations or damage to the produced cells (Storz, 2005; Ji, 2007; Kresno, 2011; Prasad et al., 2016). Aside from superoxide radicals, hydrogen peroxide may also cause cell proliferation and apoptosis. Hydrogen peroxide is also a form of non radical ROS that might act as carcinogens. Hydrogen peroxide has biphasic characteristics, because on one side it may induce apoptosis and necrosis, but on the other side it may also cause proliferation, depends on the concentration. Hydrogen peroxide at the concentration of 3-15 µM causes proliferation, at the concertation 0.5-10 mM causes apoptosis, and at the concentration of 5.0-10.0 mM it causes necrosis (Stevenson and Hurst, 2007). Meanwhile, based on the other studies, the concentration of 50 nM has been proven to stimulate cell growth in vitro in various cell pathways, such as in human intestinal cells, rat fibroblasts, and hamster fibroblasts. However, at the concentration of 1 µM-1 mM may cause cytotoxicity in cancer cells (Gupte and Mumper, 2009).

*M. malabathricum* L. fruit has a small cytotoxicity activity but a large antioxidant activity, so it is not developed as an anticancer drug, but can be developed as an antioxidant drug. Further research is needed regarding the antioxidant mechanism of *M. malabathricum* L. fruit.

In conclusion, ethanolic extract of *M. malabathricum *L. fruit contains quercetin (67.78 µg/g) and kaempferol (43.52 µg/g). The extract has antioxidant (IC50 16.82 ± 0.24 ppm) and cytotoxic activities (LC50 313.44 [283.97-344.43] ppm), which might be partly due to its quercetin content.

## Conflict of interest

The authors have no conflict of interest.
